# Molecular Phylogeny and Description of the Novel Katablepharid *Roombia truncata* gen. et sp. nov., and Establishment of the Hacrobia Taxon nov

**DOI:** 10.1371/journal.pone.0007080

**Published:** 2009-09-17

**Authors:** Noriko Okamoto, Chitchai Chantangsi, Aleš Horák, Brian S. Leander, Patrick J. Keeling

**Affiliations:** 1 Department of Botany, University of British Columbia, Vancouver, British Columbia, Canada; 2 Departments of Botany and Zoology, University of British Columbia, Vancouver, British Columbia, Canada; University of California, Riverside, United States of America

## Abstract

**Background:**

Photosynthetic eukaryotes with a secondary plastid of red algal origin (cryptophytes, haptophytes, stramenopiles, dinoflagellates, and apicomplexans) are hypothesized to share a single origin of plastid acquisition according to Chromalveolate hypothesis. Recent phylogenomic analyses suggest that photosynthetic “chromalveolates” form a large clade with inclusion of several non-photosynthetic protist lineages. Katablepharids are one such non-photosynthetic lineage closely related to cryptophytes. Despite their evolutionary and ecological importance, katablepharids are poorly investigated.

**Methodology/Principal Findings:**

Here, we report a newly discovered flagellate, *Roombia truncata* gen. et sp. nov., that is related to katablepharids, but is morphologically distinct from othermembers of the group in the following ways: (1) two flagella emerge from a papilla-like subapical protrusion, (2) conspicuous ejectisomes are aligned in multiple (5–11) rows, (3) each ejectisome increases in size towards the posterior end of the rows, and (4) upon feeding, a part of cytoplasm elastically stretch to engulf whole prey cell. Molecular phylogenies inferred from Hsp90, SSU rDNA, and LSU rDNA sequences consistently and strongly show *R. truncata* as the sister lineage to all other katablepharids, including lineages known only from environmental sequence surveys. A close association between katablepharids and cryptophytes was also recovered in most analyses. Katablepharids and cryptophytes are together part of a larger, more inclusive, group that also contains haptophytes, telonemids, centrohelids and perhaps biliphytes. The monophyly of this group is supported by several different molecular phylogenetic datasets and one shared lateral gene transfer; therefore, we formally establish this diverse clade as the “Hacrobia.”

**Conclusions/Significance:**

Our discovery of *R. truncata* not only expands our knowledge in the less studied flagellate group, but provide a better understanding of phylogenetic relationship and evolutionary view of plastid acquisition/losses of Hacrobia. Being an ancestral to all katablepharids, and readily cultivable, *R. truncata* is a good candidate for multiple gene analyses that will contribute to future phylogenetic studies of Hacrobia.

## Introduction

Katablepharids are cosmopolitan colorless flagellates that play an important role as predators in both marine and freshwater microbial ecosystems [1]–[6]. Katablepharids were originally described by Skuja [7] based on the oblong to ovate cell shape with one anterior and one posterior flagellum emerging from a subapical region. These flagellates had been classified as a subgroup of cryptophytes based on similarities observed in light microscopy, then later re-classified as *incertae sedis* based on ultrastructural studies [1]. Recent molecular phylogenetic analyses inferred from small and large subunit (SSU and LSU, respectively) rDNA sequences suggest that katablepharids are indeed a sister group of cryptophytes [8]–[11].

Although a close relationship between katablepharids and cryptophytes is clear, whether or not they are one another's closest relatives remains open to debate; several other lineages previously classified as *incertae sedis* have been shown to branch in this part of the eukaryotic tree in molecular phylogenetic analyses, such as telonemids [12], [13] and (pico)biliphytes, known only from environmental sequences and fluorescence in situ hybridization (FISH) images [14]–[17].

Their close association to cryptophytes makes katablepharids an interesting group from the perspective of the chromalveolate hypothesis. The chromalveolate hypothesis suggests that a variety of lineages that contain plastids of red algal origin (i.e., cryptophytes, haptophytes, stramenopiles, dinoflagellates, and apicomplexans) acquired them from a single common endosymbiotic event (for review, [18], [19]). Several kinds of data relating to the plastid have supported this hypothesis [20]–[23], but phylogenies based on nuclear genes have been a source of controversy [24]. The monophyly of stramenopiles and alveolates is recovered in most analyses, though with close association to non-photosynthetic rhizarians [25], [26]. Similarly, a close relationship between cryptophytes and haptophytes has also been found, predominantly in analyses based on large numbers of nuclear genes [25]–[29]. The haptophytes and cryptophytes have also been united by their unique, shared possession of a plastid *rpl36* gene derived from horizontal gene transfer [30]. Recently phylogenomic analyses have united cryptophytes and haptophytes with increasing number of non-photosynthetic lineages (e.g., [25], [29]); each new case suggests that there must have been multiple independent losses of photosynthesis in the history of this group. The clade consisting of the most recent ancestor of cryptophytes and haptophytes and all of its descendents is growing not only in diversity, but also in its importance to the chromalveolate hypothesis and the evolution of plastids. This groups has, however, yet to receive a name from the scientific community and the growing list of associated lineages has become awkwardly long; therefore, we establish the name “Hacrobia” to unite this emerging group and facilitate future discussion.

Katablepharids remain among the most poorly studied subgroups of the Hacrobia. One reason for this is that culture strains were not available until recently, and these strains require eukaryotic prey, which sets a technical challenge to purify enough material for large scale sequencing surveys. Currently, only four genera and nine species of katablepharids have been described, and molecular sequence data are restricted to small subunit ribosomal RNA (SSU rDNA) from *Katablepharis japonica*, *Leucocryptos marina* and *Hatena arenicola* [8]–[10], [31]; there are DNA sequences from an additional five genes known from *L. marina* [9]. Dozens of freshwater and marine environmental sequences are closely related to katablepharids, suggesting an unexplored diversity within this group [11]. Moreover, large-scale genomics surveys are now available for representatives of all major groups of the Hacrobia, except for the biliphytes (nearly all aspects of which remain mysterious), and katablepharids.

In this study, we report a previously undescribed, phagotrophic katablepharid inhabiting intertidal sandy beaches. We established a two-eukaryotes culture strain with a diatom as a prey source, and examined it using light and scanning electron microscopy to demonstrate the general morphology and feeding behavior of the new isolate. The cell is distinct from all other katablepharids in several ways: (1) two flagella emerge from a papilla-like protrusion in the subapical region; (2) conspicuous ejectisomes are aligned in multiple (5–11) parallel rows; (3) the size of the ejectisome is larger towards the posterior end of the rows; and (4) the cell engulfs whole prey cells within food vacuole(s). Molecular phylogenetic analyses based on heat shock protein 90 (Hsp90), small and large subunit of ribosomal RNA genes (SSU and LSU rDNA, respectively) consistently show this organism is the sister to all known katablepharids, including those known only from environmental sequences. We also used data from protein-coding genes for the first time to analyse the phylogenetic position of katablepharids relative to other lineages within the Hacrobia. Both Hsp90 and SSU consistently show a close relationship between katablepharids and cryptophytes to the exclusion of all other lineages within the Hacrobia. Nearly all of the robustly supported relationships within the Hacrobia are based on large data sets of proteins coding genes derived from genome wide surveys. A similarly large data set will almost certainly be needed to elucidate the phylogenetic position of katablepharids within the Hacrobia with confidence; however, katablepharids have been missing from such analyses due to the lack of a cultivable representative. With our description of this cultivated lineage of katablepharid, it will now be possible to acquire genomic and transcript information.

## Results

### Light microscopy

**Figure 1:**
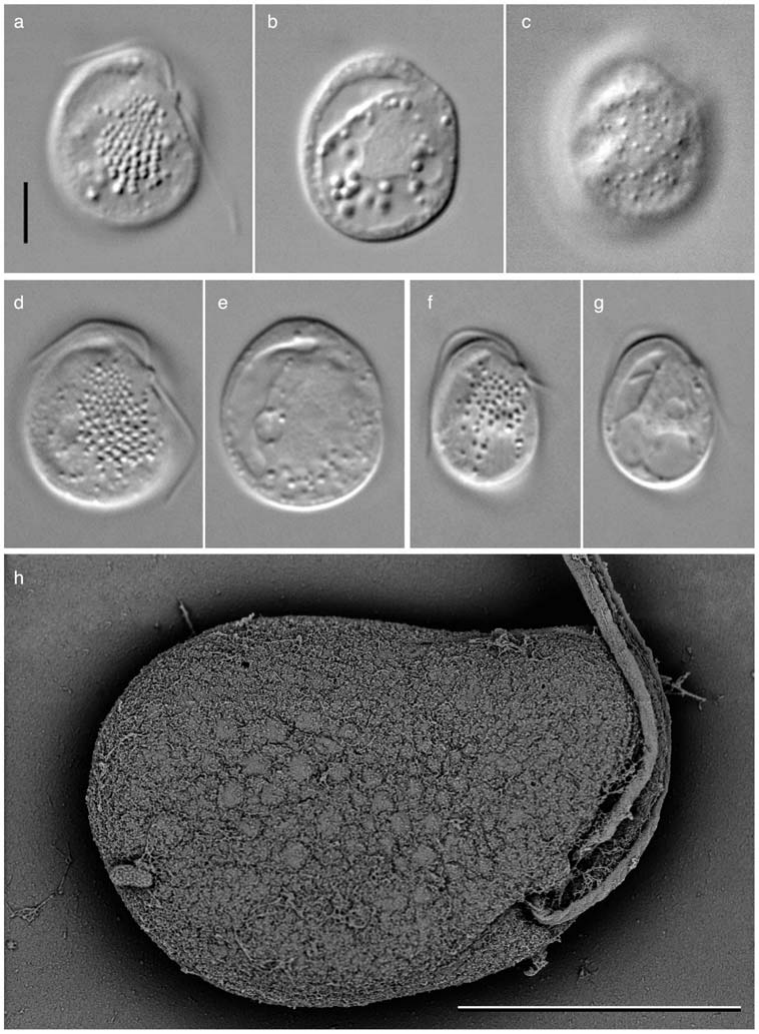
Light and scanning electron micrographs of *Roombia truncata.* sp. nov. a–c. Holotype of *R. truncata*; d–e a cell showing size close to the maximum size; f–g. a cell showing size close to the minimum size. The ventral side (a, d, f) of the cell has 5–11 rows of conspicuous ejectisomes, whose diameter ranging from c.a. 0.3 µm at the anterior end and 0.7 µm at the posterior end. Smaller ejectisomes are also present on the dorsal face of the cell (c). A cell has the anterior and posterior flagella emerging from a papilla like structure of the ventral left subapical region (a, d, f), and food vacuole along the right margin of the cell (b, e, g). (h). scanning electron micrograph showing ventral side of the cell. Note multiple rows of ejectisomes. Scale bar = 5 µm.

**Figure 2:**
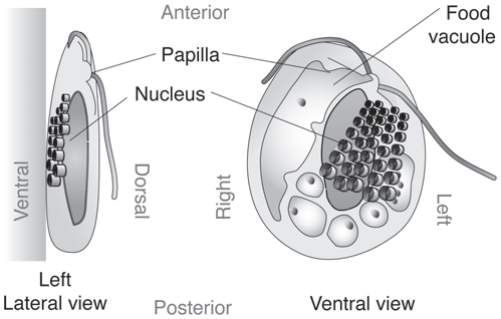
Diagram of cell structure of *R. truncata*. Left lateral view and ventral view are shown. The cell has light microscopically conspicuous ejectisomes on the ventral side. The cell glides on the surface with the ventral side down.

Figures 1 and 2 illustrate the general cell morphology of the new isolate. Cells are oval to truncated ovate in shape, dorsoventrally compressed, 12–17 µm in length and 9–14 µm in width, and lacking visible evidence of a plastid (Figures 1a–g). Two flagella emerge from a small protrusion on the left side of the subapical region of the ventral face of the cell (Figures 1a, d, f, h). The nucleus is located in the middle of the cell (Figures 1b, e, g). Most of the time the cell glides along the surface with two flagella. Although the cell occasionally comes off from the surface, it does not have a strong swimming ability.

Five to ten rows of ejectisomes are longitudinally aligned on the ventral surface of the cell. The size of the ejectisomes is gradually increased from the anterior to the posterior end of the row; i.e., 0.3 µm dia. at the anterior end and 0.7 µm dia. at the posterior end (Figures 1a, d, f, h). The largest food vacuole is located on the left margin of the cell, and a series of smaller vacuoles are located along the posterior margin of the cell.

### Feeding behavior

**Figure 3:**
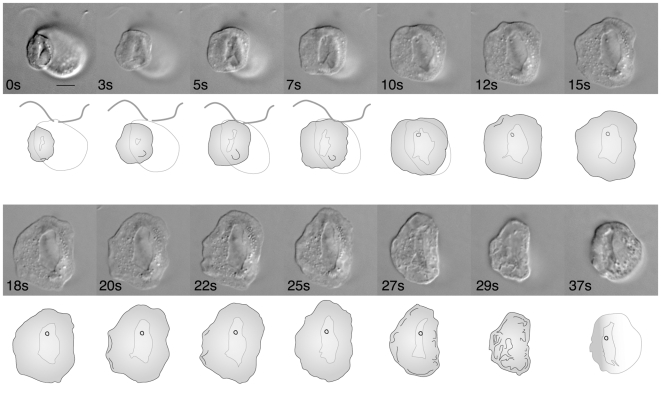
Feeding behavior on bacteria. Time in seconds from the beginning of the sequence is shown at the bottom left of each frame. The cell attaches to prey at the subapical region of the right lateral side (0 s), where the cytoplasm becomes flexible and spreads on coverslip to trap a bacterial prey cell (5 s). Once it is fully extended (25 s), the margin of the thin layer of cytoplasm thickens and contracts as the cell quickly detaches from the surface (27 s–29 s).

**Figure 4:**
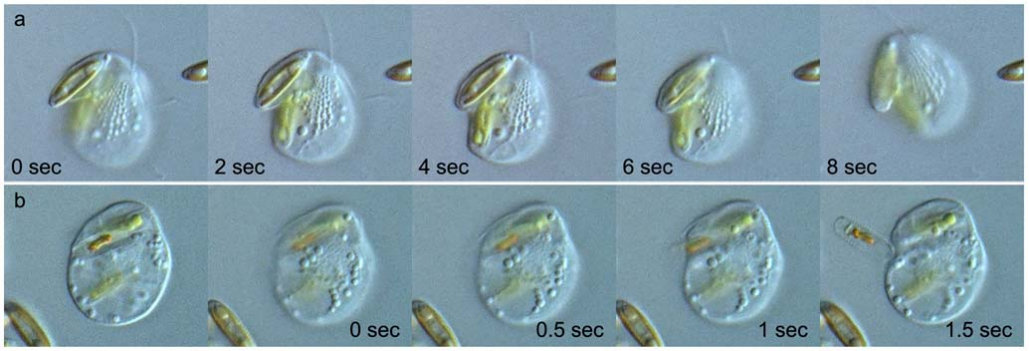
Feeding behavior on a diatom *Navicula* sp. Time in seconds from the beginning of the sequence is shown at the bottom left of each frame. (a) A series showing uptake of a *Navicula* sp. cell. (b) A series showing a process of disposing empty frustules by exocytosis after digestion. Scale bar = 5 µm.

A clonal culture of *R. truncata* (PRA-316) is maintained with *Navicula* sp. (PRA-314) and unidentified bacteria. *Roombia truncata* prefers *Navicula* sp. as a prey source, but it also feeds on the bacteria. Upon feeding, the cell attaches to a prey cell at the left anterior corner of the cell, where the cytoplasm becomes highly flexible, and then wraps around and the prey cell (Figures 3 and 4). After attaching to the coverslip, a thin layer of cytoplasm emerges from the cell, and the longest food vacuole opens to engulf the prey (Figure 3; 0–25 s). The margin of the extended cytoplasm is then thickened as it contracts to close the opening (Figure 3; 27–29 s). As the cytoplasm spreads, one may see the ventral rows of ejectisomes within the cell (e.g., Figure 3; 15–25 s).

*Roombia truncata* engulfs whole diatom cells, including the frustules (Figure 4a, Movie S1), and may take up one diatom while still digesting the previous prey diatom. After digestion is complete, *R. truncata* exocytoses the silica frustules with some pigmented debris (Figure 4b, Movie S2).

### Molecular phylogeny

**Figure 5:**
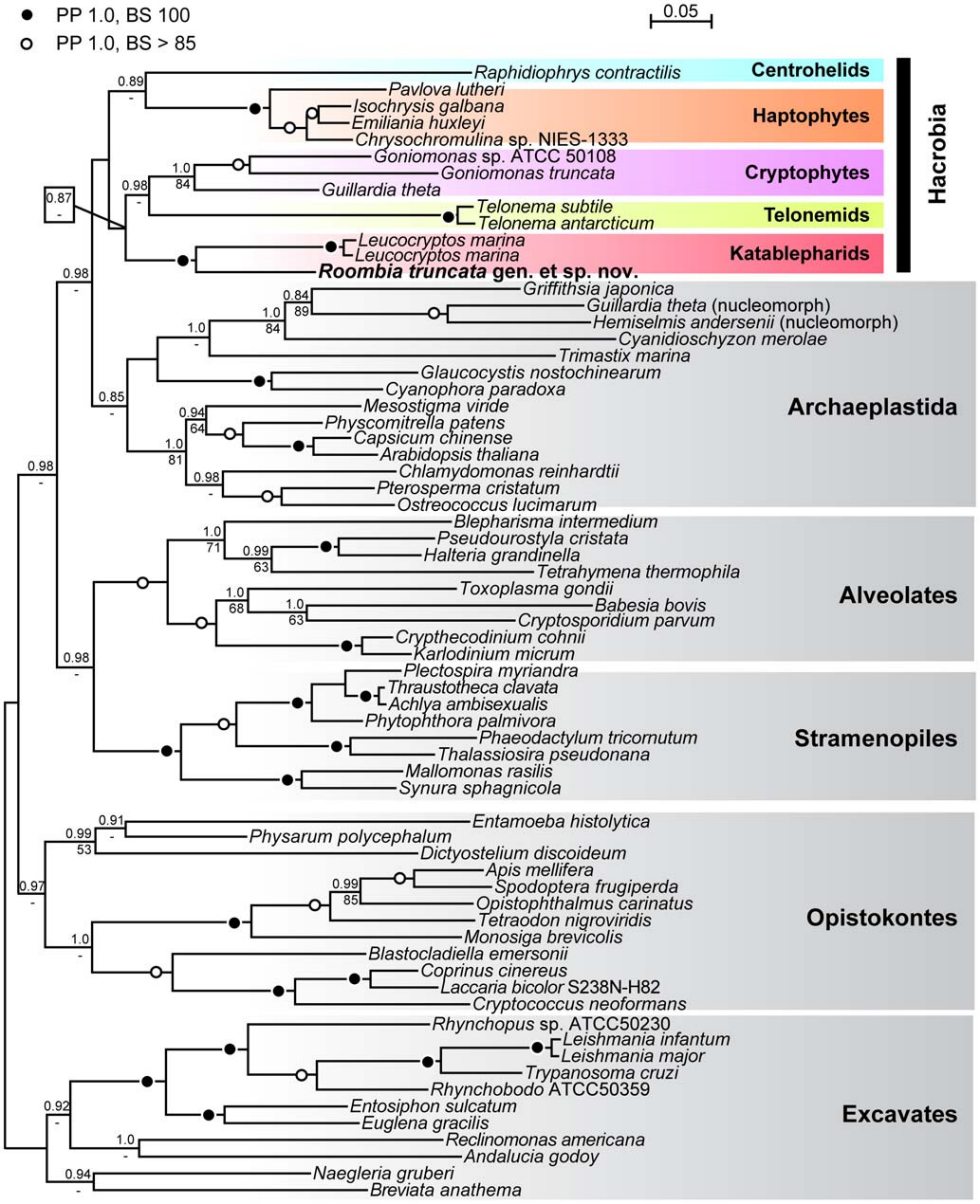
Molecular phylogeny based on Hsp90. The maximum likelihood (ML) topology based on Hsp90 sequences constructed using RTREV+GAMMA+F model of evolution. Black circles denote the branches supported by posterior probability (PP) of 1.0 and bootstrap support (BS) of 100. Open circles denote the branches supported by PP of 1.0 and BS higher than 90. BS was inferred from 1000 replications using RAxML 7.0.4, PP were assessed from 10^6^ generations with MrBayes 3.1.2 (see Methods part for details). Supergroups are boxed and shaded different colours, numbers at nodes.

We determined the DNA sequences of the SSU and LSU rRNA genes and Hsp90 in order to infer the phylogenetic position of *R*. *truncata* within the Hacrobia and more broadly examine the branching order of katablepharids and their close relatives. In all phylogenetic trees inferred from the three genes individually or combined, *R. truncata* branched with strong support as a sister lineage to katablepharids (i.e., *Leucocryptos marina* or, when other sequences were available, all katablepharid taxa; Figure 5).

**Figure 6:**
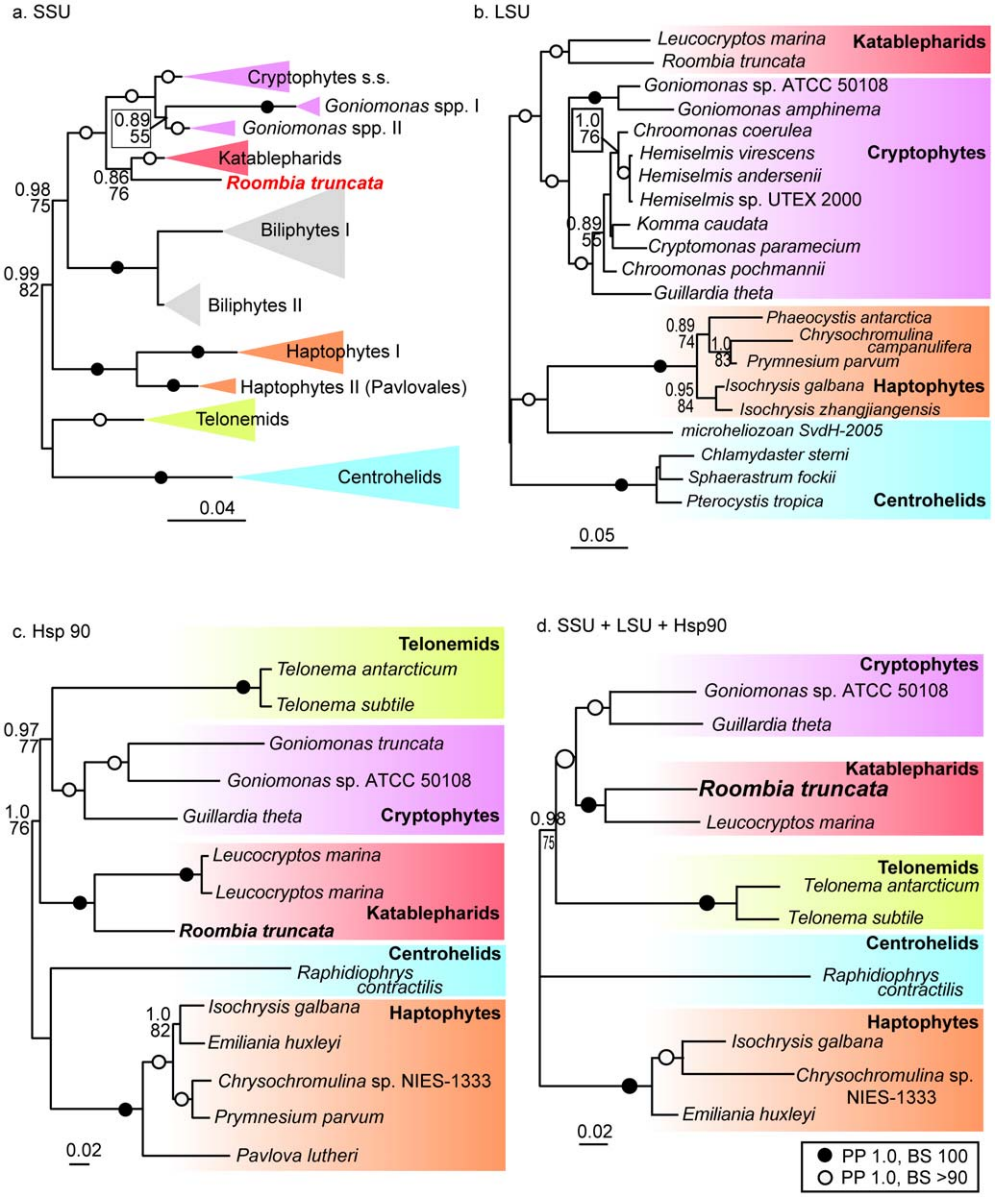
In-group analyses based on SSU, LSU, Hsp90 and combination. Datasets used were a.SSU, b.LSU, c.Hsp90, d.SSU+LSU+Hsp90, respectively. The maximum likelihood (ML) topologies were obtained using GTR+GAMMA model of evolution for rDNA sequences and RTREV+GAMMA+F model for Hsp90. Black circles denote the relationships supported by posterior probabilities (PP) of 1.0 and bootstrap support (BS) of 100%. Open circles denote the relationships supported by PP of 1.0 and BS higher than 90. BS was inferred from 1000 replications using RAxML 7.0.4, PP were assessed from 10^6^ generations with MrBayes 3.1.2 (see Methods part for details).

Katablepharids as a whole were most frequently sisters to the cryptomonads in phylogenies based on these genes (not shown), but of the genes analysed here, only the Hsp90 (Figure 5) phylogeny recovered the monophyly of most supergroups hypothesised to account for eukaryotic diversity (e.g., [29], [32], [33], including the Hacrobia (which was recovered without support). Nevertheless, many analyses have shown a clade consisting of cryptophytes and haptophytes [25]–[26], [28], [34], and they also share a common plastid horizontal gene transfer (*rpl36* [30]). Some phylogenetic analyses have also shown that centrohelid heliozoa and telonemids are related to the Hacrobia (the “CCTH group in [29]); however, it is not clear how these subgroups are related to one another and, to date, the katablepharids have not been included in any of the multigene phylogenetic analysis. Based on the fact that strong support for the monophyly of the Hacrobia has been recovered, but only when sufficient data are available, we analysed the relationships within the group using unrooted trees of Hacrobia taxa (Figure 6). In phylogenies inferred from SSU rDNA and all three genes combined (Figures. 6a, d), katablepharids form the sister group to cryptophytes to the exclusion of all other taxa. In contrast, in analyses of Hsp90 sequences (Figure 6b), telonemids form the sister group to cryptophytes, and katablepharids form the sister group to this larger group. Although no LSU rDNA sequences are available from telonemids, our results show katablepharids as the sister group to cryptophytes (Figure 6b).

In order to test alternative positions for katablepharids (specifically, *R. truncata* sp. nov.), we reduced each of the well-supported clades of the Hacrobia to two surrogate taxa. Then we constrained their monophyly, generated all possible topologies and tested them using approximately unbiased (AU) tests. In general, AU tests rejected all alternative topologies that were tested, except in the case of the SSU rDNA alignment, where AU test failed to rejected an alternative topology where *R. truncata* sp. nov. was a sister group to cryptophytes plus other katablepharids.

### Taxonomic Summary

*Roombia* gen. nov. Okamoto, Chantangsi, Horák, Leander and Keeling, 2009 (ICBN/ICZN)

urn:lsid:zoobank.org:act:7A008E1B-9FE5-42D9-920B-B58674509CEE

#### Latin description

*Cellae ovales vel oblongae truncatae secus axem dorsiventrem valde appresae, sine chromatophoro; flagellis crassis binis inaequalibus in papilla ventraliter subapicali insertis; ejectisomatibus praeditis; nucleus ad medium locatus; volans microalgas vel bacteria*.

#### Diagnosis

Cells are ovale or oblong truncate, dorsiventrally compressed, without visible evidence of plastid; two flagella emerge from a papilla-like protrusion on ventral subapical region; possessing ejectisomes; a nucleus is located in the middle; feeding on microalgae or bacteria.

Type species: *Roombia truncata*

Etymology. *Roombia* = named after Roomba(TM), a robotic vacuum cleaner (iRobot, MA) to describe its gliding motion on the surface and active feeding behavior.

*Roombia truncata* sp. nov. Okamoto, Chantangsi, Horák, Leander and Keeling, 2009 (ICBN/ICZN)

urn:lsid:zoobank.org:act:4C5EE229-68DE-4DE5-9755-827BE681CECE

#### Latin description

*Cellae ovales vel truncatae ovatae secus axem dorsiventrem valde appresae; sine chromatophoro; 12–17 *µ*m longae; 9*–*14 *µ*m latae; ventraliter subapicali cum papilla; flagellis crassis binis inaequalibus in papilla insertis; ejectisomatibus praeditis ad medium ventralis*; *ejectisomatibus anterioribus 0.3 *µ*m in diametro, ejectisomatibus posterioribus 0.7 *µ*m in diametro; nucleus ad medium locatus; vacuola digestionis ad margo dextro; vorans algam* Naviculam *sp. et bacteria*.

#### Diagnosis

Cells oval to truncated ovate, dorsoventrally compressed, 12–17 µm in length, 9–14 µm in width; lacking chromatophore; with two flagella of the same length emerging from a small papilla-like protrusion on the left ventral side of the cell; with 5–10 rows of ejectisomes on the ventral surface; anterior ejectisomes 0.3 µm in diameter, posterior ejectisomes 0.7 µm in diameter; with a nucleus in the middle; with a food vacuole along the right side of the cell; engulfing *Navicula* sp. and bacteria.

Gene sequence. A sequence of the SSU rDNA is deposited as GenBank Accession No. FJ969717.

Type locality. Blomidon Beach, Nova Scotia, Canada; longitude 64°21′7.40″W, latitude 45°15′21.13″.

Type habitat. Marine.

Data of collection: 30 July 2008

Paratypes. Figures 1a–g.

Iconotype. Figure. 2

Etymology. *Truncata* = truncated or shortened to describe the cell shape.

Cultivated material. The holotype strain is deposited in the American Type Culture Collection (ATCC, VA) as PRA-316, and the isotype strain is deposited as PRA-313. Duplicate cultures are deposited in the Microbial Culture Collection at National Institute for Environmental Sciences (NIES-MCC, Ibaraki, Japan).

Hacrobia taxon nov. Okamoto, Chantangsi, Horák, Leander and Keeling, 2009

The clade consisting of the most recent ancestor of cryptophytes and haptophytes and all of its descendents.

**Figure 7:**
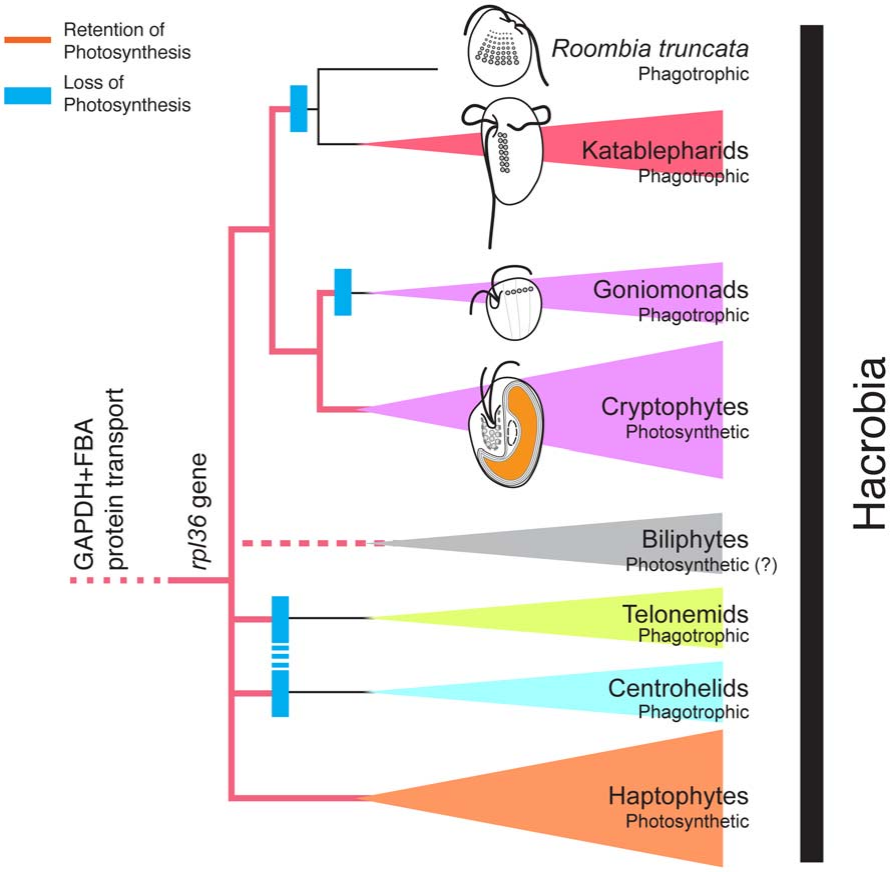
Schematic diagram of Hacrobia. Red lines denote the retention of photosynthesis. Blue boxes denote the losses of photosynthesis. If the monophyly of telonemids and centrohelids is the case as was suggested in Burki et al. [29], the number of losses of photosynthesis may be three, instead of four.

*Molecular apomorphy*: A horizontal gene transfer of the plastid *rpl36* gene, homologous to that in cryptophytes and haptophytes (Figure 7).

## Discussion

Katablepharids are heterotrophic biflagellates with oval to ovate cells that are dorsoventrally compressed, and use one anterior and one posterior flagellum to glide along substrates. When viewed with light microscopy, katablepharids are somewhat distinctive in possessing relatively thick flagella and conspicuous rows of large ejectisomes (type I ejectisomes sensu Vørs [1]). There are four described genera: *Katablepharis*, *Leucocryptos*, *Platychilomonas* and *Hatena*. *Katablepharis* spp. and *Leucocryptos marina* are planktonic with a strong swimming ability and form a noticeable swarm when feeding on smaller phytoplankton or bacteria [8], [35]. *Platychilomonas psammobia* and *Hatena arenicola* have been reported from benthic environments in the intertidal zone of sandy beaches [10], [31], [36], [37]. Although often resting on surfaces, *P. psammobia* shows similar swimming and swarming behavior to *Katablepharis* spp. and *L. marina* (Okamoto, preliminary observation), while *H. arenicola* does not swim but instead crawls on the surface of the sand and retains an *Nephroselmis*-like algal partner as a temporary phototrophic symbiont [10], [31].

In this study, we report a novel katablepharid, *Roombia truncata* gen. et sp. nov. In molecular phylogenies, *R. truncata* is sister to all currently known katablepharids (including environmental sequences). Consistent with this, *R. truncata* has several distinguishing features: (1) the papilla near the flagellar insertions, (2) distinctive ejectisomes, and (3) feeding behavior.

### Flagellar insertion

All known katablepharids have one anterior and one posterior flagellum emerging from a shallow groove or small indentation at the subapical region of the ventral right face of the cell. *Roombia truncata* has two flagella that emerge from this cellular region, but from a small protrusion, superficially similar to those seen in some green alga and cercozoans (e.g. *Protaspis* [38]). As the papilla-like structure is atypical among cryptophytes and goniomonads, it is more likely that *R. truncata* independently acquired this structure.

### Ejectisomes

Typically, katablepharids have two types of ejectisomes. Both consist of a coiled ribbon contained in a vesicle, but one is larger (710–830 nm in diameter; type I) than the other (200–300 nm in diameter; type II) [1], [35]. Type I ejectisomes are conspicuous in light microscopy and form two distinctive longitudinal rows near the flagellar insertion site on the ventral side of the cell. Type II ejectisomes are less clearly visible under light microscopy and distributed both dorsal and ventral side of the cell. *Leucocryptos marina* has an additional type III ejectisomes of different morphology and size (350–500 nm in diameter).

*Roombia truncata* possesses conspicuous ejectisomes on the ventral side, and our preliminary observation of ultrastructure suggests these are composed of the same coiled ribbon structure seen in Type I ejectisomes of other katablepharids (data not shown). However, they are aligned in the 5–11 rows, rather than the two rows typical of katablepharids. The size gradient of *R. truncata* ejectisomes within a row is also atypical: at the anterior end of a row they are about the same size of type II ejectisomes (ca 0.3 µm), increase in size so that by the posterior end of a row they are similar in size to type I ejectisomes (ca 0.7 µm). *Roombia truncata* possesses the smaller ejectisomes on the dorsal side as well.

Cryptophytes and goniomonads also have large and small ejectisomes composed of a coiled ribbon that are similar to the katablepharids type I and II ejectisomes, except that the large ejectisome of cryptophytes has a small additional coil at the distal end [39], [40].

### Feeding behavior

Katablepharids are cosmopolitan phagotrophic flagellates, feeding on both bacteria and microalgae, and play an important role in the aquatic microbial ecology both in marine and freshwater environment [1]–[6]. Ultrastructural studies have shown that katablepharids are equipped with a conical feeding apparatus at the anterior apex, consisting of numerous longitudinal microtubules lined with transverse tubular ring [1], [35], [41], superficially similar to but substantially distinct from the apical complex of alveolates. There are also numerous small, electron dense vesicles surrounded by single or double membranes associated with the feeding structure. *Katablepharis* spp., *L. marina*, and *P. psammobia* form swarms when they attack prey, attaching to small cells directly at the cell apex and then engulfing them [35], [41], or myzocytotically taking up the cytoplasm of larger prey (Okamoto, preliminary observations). In contrast, *H. arenicola* does not form a swarm, but engulfs a small prey cell without changing cell shape [10].

Interestingly, *R. truncata* appears to have a novel phagocytotic behavior. Unlike any other katablepharids, *R. truncata* flexibly expands a part or the cytoplasm to engulf the entire prey cell, even when it is a large cell (Figures 3, 4, Movie S1).

Overall, the unique features of *R. truncata* discussed above lead us to conclude it is not a member of any of the extant genera, which is consistent with our molecular phylogenetic analyses, which show *R. truncata* is a sister lineage to all other known katablepharids.

### Phylogenetic position of katablepharids within the context of the Hacrobia

While the position of *R. truncata* relative to the other katablepharids is robust, the overall phylogenetic position of katablepharids is still unsettled. It is certain that katablepharids are related to cryptophytes at some level, and are therefore included in the newly recognized group that also includes haptophytes, centrohelids, telonemids and possibly biliphytes. This group was first recognized as a clade consisting of cryptophytes and haptophytes [25]–[26], [28], [34], and sometimes referred to as the “HC” clade [34]. As taxon sampling was improved for large data sets, it was shown that this clade also includes non-photosynthetic centrohelid and telonemid protist lineages, prompting the expansion of the name to the “CCHT” group [29]. Because this group has consistent and strong support in many different analyses of different datasets, and because the acronyms currently being used to refer to this group are becoming inconsistent and unwieldy, we here established the first formal name for this group, the Hacrobia. The name is based on the names of the two main lineages that were first recognized to be related, haptophytes and cryptophytes, which also appear to span most if not all the phylogenetic diversity of the group (i.e., they are distantly related within the Hacrobia). By our definition, Hacrobia includes haptophytes, cryptophytes, katablepharids, telonemids, centrohelids, and perhaps biliphytes (pending more data from that group). Figure. 7 depicts the present membership of the Hacrobia based on this study and others [14]–[15], [17], [25]–[26], [29], [34], and our current hypothesis on the interrelationships of the Hacrobia subgroups.

Unfortunately, the phylogenetic position of katablepharids and other lineages within the Hacrobia are not decisively resolved in our analyses, although some clear hypotheses are emerging. In particular, the relative positions of katablepharids and telonemids is of interest. The topologies of Hsp90 and SSU rRNA are incompatible, but the relationship between katablepharids and cryptophytes observed in SSU trees seems the most likely, because a similar difficulties with telonemids have been observed many times. Shalchian-Tabrizi et al. [12] found that Hsp90 and concatenated Hsp90+SSU datasets support the monophyly of telonemids and cryptophytes, but single gene analyses based on SSU, LSU, alpha- and beta- tubulins genes did not. In a recent phylogenomic study, Burki et al [29] found telonemids to be a basal branch of the Hacrobia.

Morphologically, katablepharids and telonemids do not share any apparent synapomorphy, although each of them independently has some characters in common with cryptophytes. Katablepharids have morphologically similar ejectisomes as discussed above, while telonemids have mastigonemes on a single side of one of two flagella [12], [42]. Shalchian-Tabrizi et al [12] suggested that the mastigonemes of telonemids are similar to the tripartite mastigonemes of stramenopiles, in that it is comprised of three parts; a short round base, a shaft and a terminal hair. However, it is also similar to one of various types of cryptophytes mastigonemes.

Kugrens et al [43] reported a wide variety of mastigonemes within cryptophytes, of which type 5 found on *Cryptomonas caudata* seems almost identical to the mastigonemes of *Telonema subtilis* [12]; only single side of one of two flagella bears “tripartite” mastigonemes comprised of a small round base, a shaft, and a terminal hair. Although evolutionary relationships of the mastigonemes between cryptophytes, telonemids and stramenopiles are still in question, it is possible that this is an ancient character of their common ancestor.

The growing diversity of non-photosynthetic lineages recognized to belong to the Hacrobia means that photosynthesis must have been lost several times. With the exact relationships among hacrobian subgroups unknown, the number of times photosynthesis must have been lost cannot be stated, but if our hypothesis for these relationships is accurate (Figure 7), at least three losses is required. While the exact number may be unclear, the conclusion that these lineages lost photosynthesis is based on relatively strong evidence: not only do phylogenomic analyses of host genes support the monophyly of the group [25]–[29], but there is also direct evidence that the plastid was present in the ancestor of the two major photosynthetic lineages [30], which are distantly related within the group. Non-photosynthetic members of other chromalveolate groups have recently been found to contain genes derived from the plastid, and perhaps also plastids [44]–[48]. It would therefore be interesting to investigate whether non-photosynthetic members of Hacrobia also retain any such traces of a lost plastid.

### Concluding remarks

In this study, we describe a novel katablepharid, *Roombia truncata* gen. et sp. nov. and its unique phylogenetic position, morphology, and feeding behavior. Our molecular phylogenetic analyses consistently showed *R. truncata* is the sister to all hitherto known katablepharids within an emerging group of great diversity, the Hacrobia. The phylogenetic relationships within the Hacrobia are still in question, but large-scale multigene analyses have been very promising. In order to clearly determine where katablepharids fall in this group, we will obtain comprehensive genomic or transcriptomic information, a task that will be significantly aided by the ability to cultivate *R. truncata*.

### Note on International Code of Zoological Nomenclature (ICZN)

In the original description, Skuja [7] used the spelling *Kathablepharis* as a genus name, though it was grammatically incorrect. Subsequently, the genus name was corrected to *Katablepharis* under International Code of Botanical Nomenclature (ICBN), whereas under ICZN the wrong spelling *Kathablepharis* has been used (outlined by Vørs [1]). As this has caused inconvenience and confusion, we propose that the name must be corrected as *Katablepharis* under ICZN as well.

## Materials and Methods

### Strain collection, culture conditions and light microscopical observation

Surface sand samples were collected on 30 July 2008 from the intertidal zone of Blomidon Beach in the Bay of Fundy, Nova Scotia, Canada (longitude 64°21′7.40″W, latitude 45°15′21.13″). The samples were pre-cultured in f/2 or K media (Andersen et al. 2005) and kept at 18°C under the cycle of Light∶Dark = 6 h∶18 h. Subsequently, single cells were isolated by micropipetting and incubated with *Navicula* sp. (PRA-314, ATCC,VA) as a food source under the same conditions to establish the holotype strain (PRA-316, ATCC, VA) and the isotype strain (PRA-313, ATCC, VA). Light microscopy was performed with an Axioplan2 compound microscope (Zeiss, Germany) equipped with a Q imaging microimager II digital camera with a Q capture v. 2.8.1 software. Feeding and exocytosis were filmed using an XL H1s camcorder (Canon, Japan) mounted to an Axioplan2 using a PROHDVC adaptor (Micro Tech Lab, Austria) with an additional 6 mm height ring we manufactured, followed by editing on a Final Cut Express v.5 software (Apple, CA).

### Scanning electron microscopy (SEM)

Cell culture of *R. truncata* was mixed with 4% OsO_4_, giving the final concentration of 0.6% OsO_4_. The mixture was mounted on cover glasses coated by poly-L-lysine at room temperature for 30 min. The fixed samples were then washed three times in filtered f/2-Si medium to remove the fixative. The cells were dehydrated through a graded series of ethanol and critical point dried with CO_2_ using a Tousimis Samdri 795 CPD (Rockville, MD). Dried cover glasses with the fixed cells were mounted on aluminum stubs and then sputter coated with gold (5 nm thickness) using a Cressington high-resolution sputter coater (Cressington Scientific Instruments Ltd, Watford, UK). The coated cells were viewed under a Hitachi S4700 scanning electron microscope.

### Sequencing analyses

Preliminary observation revealed that most of the clonal strain of *Roombia truncata* remained on the bottom surface of the culture vessel, whereas strain PRA-316 tended to detach from the surface and float in the culture medium, which facilitates preparation of the genomic DNA with minimum contamination of *Navicula* sp. Therefore, genomic DNA of strain PRA-316 was prepared using MasterPure™ Complete DNA&RNA Purification Kit (Epicentre Biotechnologies, WI).

SSU, LSU and Hsp90 genes were amplified by nested PCR using primers listed in table S1. The PCR program was as follows: hold at 94°C for 4 min; 5 cycles of denaturation at 94°C for 30 s, annealing at 45°C for 1 min and extension at 72°C for 105 s; 35 cycles of denaturation at 94°C for 30 s, annealing at 50°C for 1 min and extension at 72°C for 105 s; and hold at 72°C for 10 min. Condition for amplification of Hsp90 was followed Kim et al [9]. Although template DNA has a minimum contamination of *Navicula* sp., sequences were determined after subcloning of PCR products to avoid the possible contamination, except in the case where katablepharids specific primers were used. Sequences were deposited in Genebank database (SSU: FJ969717; LSU: FJ969718; Hsp90: FJ969716)

### Phylogenetic analyses

The SSU and LSU rDNA sequences were aligned to the respective secondary structure based reference rDNA alignments available at http://www.arb-silva.de/download/ using Mafft 6.624 [49], [50]. The Hsp90 dataset was aligned using Mafft 6.624 and L-INS-i algorithm. Alignments were then manually edited using Bioedit 7.0.9 [51]. Sequences included in our analyses are listed in Table S2.

The maximum likelihood (ML) topologies were computed with RAxML 7.04 software [52] using GTR+GAMMA model of evolution for rDNA sequences and RTREV+GAMMA+F for HSP90. To ensure the search algorithm did not stop in a local optimum, one hundred independent runs starting with different randomized parsimony trees were performed, and the topology with highest likelihood score was chosen. Branching support was assessed using ML bootstrap analysis and bayesian posterior probability values. The bootstrap support (BS) was inferred from 1000 replications with RAxML (analysis parameters were as described above). Bayesian posterior probabilities were assessed using MrBayes 3.1.2 [53] where the Monte Carlo Markov Chain was run for 1×10^6^ generations (of which first 1×10^5^ were omitted from further reconstruction), priors were set to defaults, and model equivalents to the ML inferences were used). Combined analyses were performed using the same software and parameters.

The position of *R. truncata* was also tested using approximately unbiased (AU) test [54]. For each data set, we chose two representatives (where two or more were available) of each ingroup clade supported with 100% BS (i.e., billiphytes, katablepharids, cryptophytes, haptophytes, centroheliozoa, and telonemids), constrained their monophyly and then generated all possible topologies of these groups using PAUP 4.0b10 [55]. All topologies were then tested using the AU test as implemented in CONSEL 0.1j [56].

### Taxonomic Registration and Digital Archiving

The electronic version of this document does not represent a published work according to the International Code of Zoological Nomenclature (ICZN), and hence the nomenclatural acts contained herein are not available under that Code from the electronic edition. A separate edition of this document was produced by a method that assures numerous identical and durable copies, and those copies were simultaneously obtainable (from the publication date listed on page 1 of this article) for the purpose of providing a public and permanent scientific record, in accordance with Article 8.1 of the Code. The separate print-only edition is available on request from PLoS by sending a request to PLoS ONE, 185 Berry Street, Suite 3100, San Francisco, CA 94107, USA along with a check for $10 (to cover printing and postage) payable to “Public Library of Science.”

The online version of the article is archived and available from the following digital repositories: PubMedCentral (www.pubmedcentral.nih.gov/), and LOCKSS (http://www.lockss.org/lockss/). In addition, this published work and the nomenclatural acts it contains have been registered in ZooBank (http://www.zoobank.org/), the proposed online registration system for the ICZN. The ZooBank LSIDs (Life Science Identifiers) can be resolved and the associated information viewed through any standard web browser by appending the LSID to the prefix “http://zoobank.org/”.

The ZooBank LSID for this publication is urn:lsid:zoobank.org:pub:538D5A28-1B4D-4F17-9698-A18A08F9ED5C.

## Supporting Information

Table S1 Primers used in this study. Primers used in this study and references are listed below. S: sense direction; AS: antisense direction.
(0.06 MB DOC)

Table S2 Accession numbers of the sequneces used in this study. Accession numbers of the sequences used in this study are shown.
(0.12 MB XLS)

Movie S1 Feeding process of *Navicula* sp. *Roombia truncata* cells feeding on *Navicula* sp. are shown.
(4.27 MB MOV)

Movie S2 Disposing process of empty frustules of *Navicula* sp. *Roombia truncata* cells disposing empty frustules of *Navicula* sp. by exocytosis after digestion is shown.
(2.30 MB MOV)
