# Supplementary material for: Molecular Phylogeny and Description of the Novel Katablepharid Roombia truncata gen. et sp. nov., and Establishment of the Hacrobia Taxon nov
Source: PLoS One. 2009 Sep 17;4(9):e7080. doi: 10.1371/journal.pone.0007080 (PMC2741603; doi:10.1371/journal.pone.0007080)
Supplement: Table S1 — Primers used in this study. Primers used in this study and references are listed below. S: sense direction; AS: antisense direction. (0.06 MB DOC) [file pone.0007080.s001.doc]

**Supplemental table 1. Primers used in this study.**

Primers used in this study and references are listed below. S: sense direction; AS: antisense direction.

| Primer | direction | Sequence | Reference |
| --- | --- | --- | --- |
| SSU |  |  |  |
| NPF1 | S | 5'-TGCGCTACCTGGTTGATCC-3' | Chantangsi and Leander 2009 |
| 525F | S | 5'-AAGTCTGGTGCCAGCAGCC-3' | Hoppenrath et al 2007 |
| 1050MRD | AS | 5'-GCCTYGCGACCATACTCC-3' | Chantangsi and Leander |
| FAD | AS | 5'-TGATCCTTCTGCAGGTTCACCTAC-3' | ‘B’ in Medlin et al 1998 |
|  |  |  |  |
| LSU |  |  |  |
| 28S-1F | S | 5'-ACC CGC TGA ATT TAA GCA T-3' | Moreira et al (2007) |
| 28S-568F | S | 5'-TTG AAA CAC GGA CCA AGG AG-3' | Moreira et al (2007) |
| LSU-Kata-F | S | 5'-CAC CKC ACG TCT TGM GGT GTC-3' | This study |
| LSU-Kata-R | AS | 5'-GAC ACC KCA AGA CGT GMG GTG-3' | This study |
| 28S-2F | S | 5'-GCA GAT CTT GGT GGT AG-3' | Moreira et al (2007) |
| 28S-2R | AS | 5'-CTM CCA CCA AGA TCY GC-3' | Chantangsi in prep. |
| 28S-1611R | AS | 5'-CTT GGA SAC CTG MTG CGG-3' | Moreira et al (2007) |
| 28S-3R | AS | 5'-CAC CTT GGA GAC CTG CT-3' | Moreira et al (2007) |
| 28S-4R | AS | 5'-TTC TGA CTT AGA GGC GTT CAG-3' | Moreira et al (2007) |
|  |  |  |  |
| Hsp90 |  |  |  |
| 100XF | S | 5'-CAG CTG ATG TCC CTG ATC ATY AAY CAN TTY TA-3' | Simpson et al (2002) |
| HspFC | S | 5'-TSA AGG ACC TSR TCA AGA AGC A-3' | Kim et al (2006) |
| HspRD | AS | 5'-CTC NCC RGT GAT GWA GTA GAT-3' | Kim et al (2006) |
| 910XR | AS | 5'-TCG GGG TTG ATY TCC ATN GTY TT-3' | Simpson et al (2006) |

Chantangsi, C, Leander BS (2009) An SSU rDNA barcoding approach to the diversity of marine interstitial cercozoans, including descriptions of four new genera and nine new species. Int J Syst Evol Microbiol *in review*

Moreira D, von der Heyden S, Bass D, Lopez-Garcia P, Chao E, Cavalier-Smith T. 2007. Global eukaryote phylogeny: Combined small- and large-subunit ribosomal DNA trees support monophyly of Rhizaria, Retaria and Excavata. Mol Phylogenet Evol. 44:255-266.

Hoppenrath M, Horiguchi T, Miyoshi Y, Selina M, Taylor MFJR, Leander BS (2007) Taxonomy, phylogeny, biogeography, and ecology of *Sabulodinium undulatum* (Dinophyceae), including an emended description of the species. Phycol Res. 55(2):159–175

Medlin L, Elwood HJ, Stickel S, Sogin ML. (1988) The characterization of enzymatically amplified eukaryotic 16S-like rRNA-coding regions. Gene 71:491-499.

Simpson AGB, Lukes J, Roger AJ. 2002. The evolutionary history of kinetoplastids and their kinetoplasts. Mol Biol Evol. 19:2071–2083.

Simpson AGB, Inagaki Y, Roger AJ. 2006. Comprehensive multi-gene phylogenies of excavate protists reveal the evolutionary positions of “primitive” eukaryotes. Mol Biol Evol. 23: 615–625.
